# Chemistry and Pathology

**Published:** 1897-09-18

**Authors:** 


### CHEMISTRY AND PATHOLOGY.

In his address at Moscow, Dr. Lauder Brunton showed that the link by which the sciences of physiology, pathology, and pharmacology are bound together is chemistry; and as the performances of every cell must depend largely upon the nature of the medium in which it lives, and from which it derives both its nourishment and its means of doing work, it is easy to see that this must be the case. That being granted, he proceeded to show that many of the processes of pathology and even of physiology differ from those observed in the study of pharmacology only in the fact that the poisons and ferments which produce them are formed within the body instead of being taken from outside.

The *rapprochement* between these different branches of science is, indeed, taking place from both sides; while, on the other hand, the pathological importance of microbes is cast into the shade by the interest attaching to their products—the drugs they brew—by means of which the symptoms of disease are caused; on the other hand, pharmacology has ceased to confine itself to the investigation of alkaloids and other final products of vegetable life or of the laboratory, and concerns itself now with animal juices directly formed by animal cells reacting under the irritation of various stimuli. Whether, then, we deal with questions of physiology, such as digestion; of pathology, such as the pyrexia and other symptoms produced by the germs of the specific fevers, or the hyperplasia caused by the bacilli of tuberculosis; or again, of pharmacology, as in the treatment of heart disease with digitalis, or of syphilis with mercury; in each case we have to do with questions which in essence depend on certain changes in the chemical constitution of the pabulum of the cell.

It is thus to physiological and pathological chemistry, in other words to pharmacology, that we must look for further advance in practical medicine. "Practical medicine," says Lauder Brunton, "depends on physiology, pharmacology, and pathology, but all three are tending to become more and more subdivisions of the wider and all embracing science of chemistry." Yes! and when chemistry shall have become so all embracing as to explain life, which is the problem left for the next century to solve, then indeed chemistry will be the master science. In the meantime the chemistry of the body differs from that of the test tube, the living cell stands between the reagent and the reaction, and according to the quality and degree of "life" within that cell so does the product vary.

